# Quorum Regulated Resistance of *Vibrio cholerae* against Environmental Bacteriophages

**DOI:** 10.1038/srep37956

**Published:** 2016-11-28

**Authors:** M. Mozammel Hoque, Iftekhar Bin Naser, S. M. Nayeemul Bari, Jun Zhu, John J. Mekalanos, Shah M. Faruque

**Affiliations:** 1 Laboratory Sciences and Services Division, International Centre for Diarrhoeal Disease Research, Bangladesh, Dhaka-1212, Bangladesh; 2 Department of Microbiology, Perelman School of Medicine, University of Pennsylvania, 3610 Hamilton Walk, Philadelphia, 19104-6076 USA; 3 Department of Microbiology and Immunobiology, Harvard Medical School, 77 Avenue Louis Pasteur, Boston, MA 02115, USA

## Abstract

Predation by bacteriophages can significantly influence the population structure of bacterial communities. *Vibrio cholerae* the causative agent of cholera epidemics interacts with numerous phages in the aquatic ecosystem, and in the intestine of cholera patients. Seasonal epidemics of cholera reportedly collapse due to predation of the pathogen by phages. However, it is not clear how sufficient number of the bacteria survive to seed the environment in the subsequent epidemic season. We found that bacterial cell density-dependent gene expression termed “quorum sensing” which is regulated by signal molecules called autoinducers (AIs) can protect *V. cholerae* against predatory phages. *V. cholerae* mutant strains carrying inactivated AI synthase genes were significantly more susceptible to multiple phages compared to the parent bacteria. Likewise when mixed cultures of phage and bacteria were supplemented with exogenous autoinducers CAI-1 or AI-2 produced by recombinant strains carrying cloned AI synthase genes, increased survival of *V. cholerae* and a decrease in phage titer was observed. Mutational analyses suggested that the observed effects of autoinducers are mediated in part through the quorum sensing-dependent production of haemaglutinin protease, and partly through downregulation of phage receptors. These results have implication in developing strategies for phage mediated control of cholera.

Toxigenic *Vibrio cholerae*, the causative agent of the epidemic diarrhoeal disease cholera interacts with numerous bacteriophages both in the aquatic environment and inside the human intestine^1^,^2^,^3^. Phages which infect *V. cholerae* (vibriophages) also contribute to the evolution of this pathogen by mediating horizontal transfer of genes and genomic rearrangements^4^,^5^,^6^,^7^. Cholera epidemics are known to be self-limiting in nature, since the epidemics subside after reaching a peak, even without any active human intervention. Among other factors, lytic phages that kill *V. cholerae* have been shown to play a significant role in modulating the course of epidemics presumably through their inherent bactericidal activity^1^,^2^. In this latter process, bacterial mutants that are able to resist phage predation (for example, those that have lost cell surface receptors required for phage infection) presumably enjoy a survival advantage. However, phages frequently utilize receptors that are crucial to the pathogenicity of *V. cholerae* such as cell surface lipopolysaccharide O side chain polysacchardies^8^. Furthermore, an antagonistic interaction between a *V. cholerae* chromosomal island that encode phage resistance, and phage encoded CRISPR (clustered regularly interspaced short palindromic repeats) that attacks the island’s DNA sequence, provides an additional example of the ‘arms race’ that occurs between *V. cholerae* and its phages^9^.

In the aquatic environment *V. cholerae* is known to exist mostly as biofilms which are comprised of densely packed cells embedded in an exopolysaccharide matrix, or as aggregates of dormant cells referred to as conditionally viable environmental cells (CVEC)^10^,^11^. Likewise, the colonization of the human gut by *V. cholerae* leads to a state of high bacterial cell density in the intestinal lumen and mucosal surface. The stools of cholera victims are replete with clumps of *V. cholerae* suggesting that bacterial cells may indeed interact closely with each other at high densities during the latter stages of the infection cycle^11^. Independent evidence for cell-cell interactions *in vivo* has also been obtained in a *V. cholerae* animal model through Tn-seq mutational analysis^12^.

Gene expression dependent on bacterial cell density referred to as “quorum sensing” is known to regulate metabolic processes that may influence bacterial survival under unfavourable conditions^13^,^14^,^15^,^16^. The regulatory pathways which control cell density dependent metabolic responses in *V. cholerae* include two autoinducers (CAI-1 and AI-2) and their cognate receptors CqsS and LuxPQ respectively, along with a signal transduction cascade that involves phosphorylation and de-phosphorylation of transcriptional regulatory proteins, non-coding small RNAs, and RNA chaperons^17^,^18^,^19^. A recent study has proposed the existence of two additional autoinducer sensors in *V. cholerae*, namely VpsS and CqsR, but the signals sensed by these sensors are presumably different from the two canonical autoinducers, CAI-1 and AI-2^20^. Since the density of bacterial population may be a risk parameter for increased exposure to phages, in this study we investigated whether quorum sensing could modulate sensitivity of *V. cholerae* to phage predation. Our results suggest that quorum sensing does indeed modulate the sensitivity of *V. cholerae* to phage infection through several ways that include extracellular phage inactivation by haemagglutinin protease (HAP) as well as modulation of the function or accessibility of phage to the LPS O-antigen receptor.

## Results

### Autoinducers alter phage-bacterial growth kinetics

**Figure 1:**
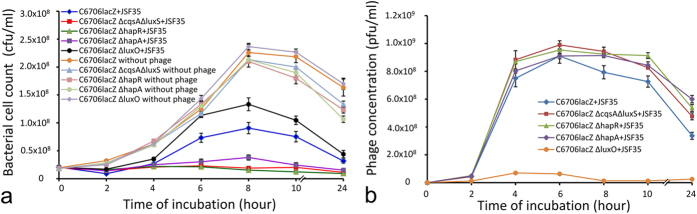
Phage-bacterial growth kinetics in mixed cultures of a phage and a susceptible *V. cholerae* O1 strain, C6706*lacZ* or its derivatives. LB medium was inoculated simultaneously with a laboratory-grown bacterial culture and the phage preparation (diluted to a concentration of ~10^7^ bacteria and 10^6^ phage particles per ml), and incubated at 37 °C with shaking. Samples were then removed at regular intervals and analyzed for the presence of phage and *V. cholerae* using standard plating techniques. (**a**) Growth curve of *V. cholerae* strain C6706*lacZ* and its derivatives carrying mutations in quorum sensing associated genes in the presence of a lytic phage JSF35 as indicated; (**b**) Titer of phage JSF35 subjected to different growth conditions and host strains as indicated. Each data point represents the mean and standard deviation of three observations.

To examine whether autoinducers CAI-1 and AI-2 enhance the resistance of bacteria against lytic phages, we monitored the kinetics of phage and bacterial growth in mixed cultures of a defined phage and a *V. cholerae* strain C6706*lacZ* or its isogenic mutants carrying inactivated autoinducer synthase genes. As shown for phage JSF35 (Fig. 1), the parent bacterial strain was found to survive and multiply more efficiently than the autoinducer negative mutant strain in the presence of the phage (Fig. 1a). This phenomenon was also reflected in the growth kinetics of the phage, as the phage amplified more rapidly when cultured with the mutant strains as compared to the parent strain (Fig. 1b).

With a comparable initial count of the mutant and parent bacterial strains, by 8 hours of growth in the presence of the phage, the mean count of the parent strain reached ~4.8 fold higher than the mutant strain (p = 0.0055) which carried inactivated AI synthase genes. There was no significant difference between the growth rate of the two strains in the absence of the phage during the same period of incubation. In addition, *V. cholerae* carrying mutations in genes involved in quorum regulation (*luxO, hapR* or *hapA*) also showed marked alteration in their growth kinetics in the presence of the phage as compared to that of the parent strain (Fig. 1). Notably, the growth curves of these mutant strains in the absence of the phage were similar to that of the parent strain during the first 8 h of growth; beyond this time the cell counts of the *hapR* or *hapA* negative strains declined somewhat more rapidly than the parent strain (Fig. 1), whereas that of the *luxO* negative mutant was found to remain comparable to that of the parent strain.

**Figure 2:**
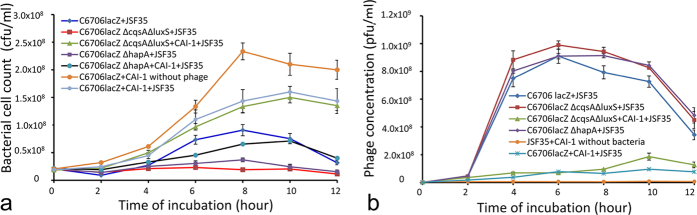
Effect of an exogenous autoinducer on phage-bacterial growth kinetics in mixed cultures of a phage and a susceptible *V. cholerae* O1 strain C6706*lacZ* or its derivatives. LB medium was supplemented with 50% (v/v) spent medium of recombinant *E. coli* over-producing or not producing the *V. cholerae* autoinducer CAI-1. The medium was inoculated simultaneously with a laboratory-grown bacterial culture and the phage preparation (diluted to a concentration of ~10^7^ bacteria and 10^6^ phage particles per ml), and incubated at 37 °C with shaking. Samples were then removed at regular intervals and analyzed for the presence of phage and *V. cholerae* using standard plating techniques. Appropriate control assays were run in parallel to monitor the stability of the phage in the spent culture supernatants in the absence of the host strain. (**a**) Growth curve of *V. cholerae* strain C6706*lacZ* and its derivatives with a lytic phage JSF35 in the presence or absence of exogenous *V. cholerae* autoinducer CAI-1 as indicated; (**b**) Titer of phage JSF35 subjected to different growth conditions and host strains as indicated. Each data point represents the mean and standard deviation of three observations.

We further studied kinetics of phage and bacterial growth in mixed cultures of phage JSF35 and *V. cholerae* strain C6706*lacZ* supplemented with various spent culture medium from recombinant *E. coli* carrying cloned *V. cholerae* autoinducer synthase genes which could be expressed under an IPTG inducible *lac* promoter^17^. We found that addition of 50% (v/v) spent medium from IPTG-induced *E. coli* DH5α cultures expressing cloned CAI-1 or AI-2 synthase genes showed a marked alteration in the phage bacterial growth kinetics in favour of the bacteria (Fig. 2; see Supplementary Fig. S1). Supplementation with culture supernatants containing CAI-1 caused the AI synthase deficient mutant strain to grow to a ~6.9 fold higher count compared to the same strain grown without CAI-1 supplementation (p = 0.0039) by 8 h of incubation. This effect was not observed when the mixed culture was grown in an identical manner but exposed to spent culture media of *E. coli* DH5α carrying the empty cloning vector pTAC, or that of *E. coli* carrying cloned autoinducer synthase genes, but grown in the absence of IPTG which controlled their expression (data not shown).

### Autoinducers CAI-1 and AI-2 diminish phage susceptibility of *V. cholera*

**Figure 3:**
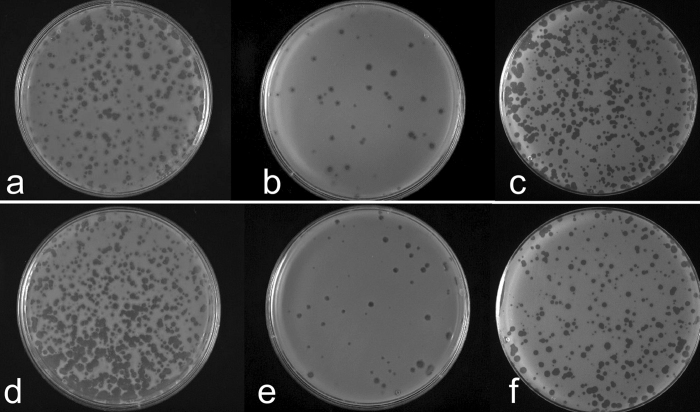
Plaques produced by bacteriophage JSF35 on a lawn of *V. choleare* O1 strain C6706*lacZ* and its derivatives grown under different conditions. Logarithmic phase cells grown in LB supplemented with 50% (v/v) spent culture medium from recombinant *E. coli* strains over-expressing or not expressing cloned genes for CAI-1 or AI-2 synthesis were mixed with a known titer of the phage in soft agar, and overlaid on LB agar plates. Plaques produced on lawns of the bacteria grown in medium containing spent culture supernatants of IPTG induced recombinant *E. coli* strains producing CAI-1 (**b**) or AI-2 (**e**), and control plates (**a,d**) with plaques formed on indicator strain grown in medium supplemented with spent culture medium of the respective recombinant *E. coli* strains without IPTG-induction are shown. Derivatives of strain C6706*lacZ* lacking receptors for CAI-1 or AI-2 grown under similar conditions as parent strain C6706*lacZ* and used in these assays are also shown (**c,f** respectively). This set of controls was used to ascertain that the observed effect of added spent culture media on phage plaque counts was indeed due to the respective autoinducers present in the spent media.

We further used soft agar plaque counts on a lawn of the target host bacteria to directly examine the effect of autoinducers CAI-1 and AI-2 on phage-susceptibility after the bacteria were grown in the presence of exogenous autoinducers added to the culture medium. An overnight culture of the host *V. cholerae* strain C6706*lacZ* was diluted 100-fold into fresh LB supplemented with 50% (v/v) spent culture medium of recombinant *E. coli* strains over-expressing or not expressing cloned CAI-1 or AI-2 synthesis genes, and was grown to log phase. These cultures were used to test susceptibility of the bacteria to various phages by using the quantitative plaque assay. We found that pre-treatment of the *V. cholerae* strain in LB supplemented with 50% (v/v) spent culture medium containing CAI-1 or AI-2 markedly reduced the plaque count yield compared to appropriate control cultures under identical conditions of assay (Fig. 3 and Table 1). This effect was not observed when the indicator strain grown under similar conditions but in LB supplemented with spent culture medium of *E. coli* carrying the empty cloning vector was used (Table 1). Similarly, there was no observable difference in plaque counts when culture supernatants of the respective recombinant *E. coli* strains without IPTG induction were used to supplement growth medium of the *V. cholerae* indicator strain (Fig. 3). To further confirm that the observed effect of added culture supernatants in reducing phage plaque counts was indeed due to autoinducers, we conducted a set of similar assays using mutant strains (C6706*lacZ*, *cqsS*::TnFGL3 and C6706*lacZ*, *luxP*::TnFGL3) which carried transposon insertions in the gene encoding a receptor for the respective autoinducer (Supplementary Table S1). Mutants carrying inactivated *cqsS* or *luxP* gene did not exhibit the reduction in phage plaque counts in response to added culture supernatant of the recombinant *E. coli* strain containing the corresponding autoinducer (Fig. 3).

### Quorum induced hemagglutinin protease inactivates vibriophages

We further examined the role of three genes (*luxO*, *hapR* and *hapA*) by observing whether phage susceptibility was altered in mutant strains with inactivation of these regulatory loci. We found that inactivation of *luxO* caused a dramatic reduction in phage susceptibility of the indicator strain (p = 0.0003), and this change in susceptibility did not depend on the presence or absence of autoinducers in the culture medium (Table 1). Since the literature predicts that a *luxO* mutant should over express hemamglutinin protease (HAP) encoded by *hapA* which might inactivate the phage, we further examined this penomenon in a *luxO* and *hapA* double mutant. The reduced phage susceptibility of the luxO negative derivative was found to be partially reversed in a double *luxO* and *hapA* mutant, but still remained significantly lower than the parent strain C6706*lacZ* (p = 0.02), suggesting that HAP was only partly responsible for the observed reduced phage susceptibility of the *luxO* mutant. We also tested whether culture supernatants of various strains contained phage-inactivating activity that depended on *hapA*. We thus exposed diverse phages to various spent culture supernatants including those of the *luxO* mutant and *luxO hapA* double mutant for 6 h to detect anti-phage activity, and determined residual phage titer after 6 h incubation (Supplementary Table S2). All 15 phages tested were shown to be affected by varying degrees and the observed phage stability ranged from 34% to 95% under various conditions specified (Supplementary Table S2) with lowest stability under conditions when HAP was over expressed.

### Quorum sensing enhances the emergence of phage resistant *V. cholerae*

**Figure 4:**
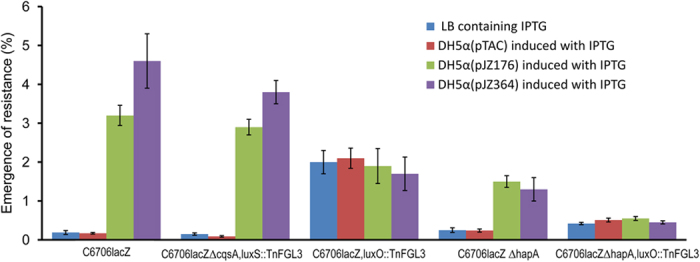
Effect of autoinducers on the emergence of phage resistant derivatives of *V. cholerae* O1 strain C6706*lacZ* and its defined mutants. Each bacterial strain was grown in LB media supplemented with IPTG-induced spent culture media of recombinant *E. coli* strains expressing or not expressing cloned autoinducer synthase genes, before estimating the emergence of resistant derivatives by exposing to phages. Values shown are mean and standard deviations of 3 different observations using phage JSF35. The rate of emergence of phage resistant derivatives was higher when the bacterial strain was grown in LB supplemented with spent medium of *E. coli* DH5α (pJZ176) containing CAI-1 (p = 0.0025) or *E. coli* DH5α (pJZ364) containing AI-2 (p = 0.0077) as compared to that of the strain grown in LB supplemented with spent medium of *E. coli* DH5α carrying the empty cloning vector (pTAC). The *hapA* negative strain also showed significant difference in the rate of emergence of phage resistant derivative when grown in medium containing CAI-1 (p = 0.0029) or AI-2 (p = 0.0196) as compared to that grown in LB containing spent medium of *E. coli* DH5α (pTAC).

To determine whether in addition to production of HAP, possible other quorum-mediated changes in the bacteria were also responsible for the observed phage resistance, we compared the rate of emergence of resistant derivatives among *V. cholerae* strain C6706*lacZ* and its various mutants when cultured in the presence or absence of exogenous autoinducers (Fig. 4). Using an assay developed by us previously^21^, we quantified the frequency of emergence of phage resistant mutants, and found that growth in LB supplemented with spent medium containing autoinducers caused the emergence of phage resistant derivatives of the target bacterium at an enhanced rate as compared to a culture without added autoinducer preparations (Fig. 4). The rate of emergence of phage resistant derivatives of strain C6706*lacZ* was significantly higher when strain was grown in medium supplemented with IPTG-induced culture supernatant of *E. coli* DH5α (pJZ176) producing CAI-1 (p = 0.0025) or *E. coli* DH5α (pJZ364) producing AI-2 (p = 0.0077) as compared to that of the strain grown in LB containing IPTG induced spent culture media of *E. coli* DH5α carrying the empty cloning vector pTAC. The *hapA* negative strain also showed a significant difference in the rate of emergence of phage resistant derivative when the strain was grown in medium supplemented with IPTG-induced culture supernatant of *E. coli* DH5α (pJZ176) producing CAI-1 (p = 0.0029) or *E. coli* DH5α (pJZ364) producing AI-2 (p = 0.0196) as compared to that of the strain grown in LB containing IPTG induced spent medium of DH5α carrying the empty cloning vector.

The *luxO* mutant showed enhanced emergence of phage resistant derivatives compared to the parent strain irrespective of whether the strain was grown in the presence of an autoinducer or not (Fig. 4). Representative phage resistant colonies were picked from the primary plates, grown and retested for resistance to the same phage again, and all but two of 270 such colonies tested were found to maintain the resistance phenotype. Representative resistant colonies were also tested for agglutination with *V. cholerae* O1 serotype specific antiserum. We found that approximately 90% of the colonies picked had lost their ability to agglutinate with O1 specific antiserum. Extensive sub-culturing of the phage resistant derivatives failed to reinstate phage sensitivity or the O1 serotype except for two phage resistant derivatives noted above, which became phage susceptible and regained the ability to agglutinate with the O1 antiserum. The reasons for this apparent stable phage resistance were not clear except that most of these variants lost the ability to express the O1 antigen.

### O-antigen gene mutants of *V. cholerae* are resistant to multiple phages

Many vibriophages are known to use the bacterial LPS/O-antigen to bind to the cell surface of *V. cholerae*^8^. Since most of the phage resistant colonies in the present study lost the ability to agglutinate with O1 specific antiserum, we verified whether the O-antigen was indeed a receptor for some of the 15 vibriophages used in this study. To do this, we screened a defined transposon insertion library of a host *V. cholerae* strain C6706*lacZ*^22^ and identified mutants that were resistant to one or more of the phages. In this screen, we found that all mutants that were phage resistant carried transposon insertion in one or more genes associated with O-antigen biosynthesis. Mutants carrying transposon insertion in 9 of 11 genes of the O-antigen biosynthetic gene cluster screened in this assay showed resistance to one or more phages (Supplementary Table S3). Collectively, these data suggest that exposure to AI in the experiments described earlier in this report may reduce or eliminate O1 antigen expression through either genetic (e.g., phase variation or mutation) or phenotypic modulation (e.g., transcriptional or epigenetic changes). These changes in O1 antigen expression could explain the observed alterations in phage replication and emergence of phage resistant mutants.

### Autoinducers affect adsorption of phages to *V. cholerae* cells

**Figure 5:**
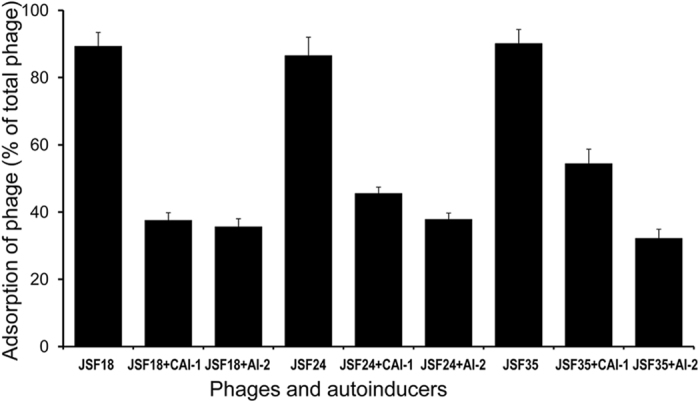
Adsorption of different phage strains to *V. cholerae* O1 strain C6706*lacZ* which was pre-treated in the presence or absence of autoinducers by supplementing the culture medium with 50% vol:vol spent culture supernatants of recombinant *E. coli* DH5α producing *V. cholerae* CAI-1 or AI-2 (see methods for details). To examine the adsorption of the phage to indicator bacterial cells, ~2.5 × 10^8^ cells in the broth culture of the *V. cholerae* strain was mixed with 10^7^ pfu of the phage. Parallel samples and appropriate controls were incubated for 20 min at 30 °C. The concentrations of non-adsorbed phages were determined by separating the free phages from the culture and conducting plaque assays. Adsorption of different phages to the bacterial cells (percentage of total phage particles used) and the corresponding growth condition of the host bacteria are shown.

An essential step in the susceptibility of bactreria to a phage is the the adsoprtion of phage particles on the bacterial surface through specific receptors. In an attempt to investigate the mechanisms associated with altered suceptibility of *V. cholerae* strains to phages, adsorption of different phages to *V. cholerae* strain C6706*lacZ* was studied after pre-growth of the bacteria in the presence and absence of added autoinducer preparations. A total of 15 different phages to which the host bacterial strain C6706*lacZ* was susceptible were tested. As shown in Fig. 5, there was a distinct effect of autoinducers on absorption of phages, with adsorption being diminished significantly when the bacteria were pretreated in autoinducer preparations. This observation suggests that the diminished susceptibility of *V. cholerae* to phages in the presence of autoinducers occurred at the level of phage-adsorption.

### Autoinducers CAI-1 or AI-2 down-regulate the expression of O-antigen biosynthetic genes

**Figure 6:**
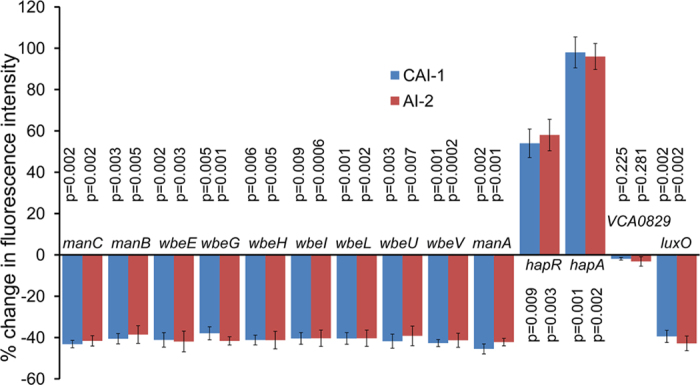
Effect of autoinducer CAI-1 and AI-2 on the expression of O-antigen biosynthetic genes. GFP fusion constructs of relevant genes were grown in LB medium in the presence or absence of added autoinducer preparations. The percentage increase or decrease in fluorescence intensity per unit cell density are shown. Values represent the mean and standard deviations of three independent observations. GFP fusions to known quorum regulated genes *luxO*, *hapR* and *hapA,* and a gene designated *VCA0829* encoding Acetyl CoA synthase which is not regulated by autoinducers were used as controls. Two tailed p values for the difference in decrease or increase in expression of different GFP fusion constructs when grown in media containing exogenous autoinducers compared to that without added autoinducers are shown. There was no significant difference in expression of *VCA0829* under these conditions.

We examined whether expression of the O-antigen biosynthetic genes were altered when the host strain C6706*lacZ* was grown in the presence of 50% vol:vol spent medium from *E. coli* DH5α cultures expressing cloned CAI-1 or AI-2 by monitoring the expression of a number of such genes. In this assay we used derivatives of strain C6706*lacZ*, carrying GFP fusions in the O-antigen genes, due to the presence of GFPmut3 gene in TnFGL3 used in construction of the transposon insertion library^22^. The expression of GFP was measured by fluorometry, to determine the effect of exogenous autoinducers on expression of 10 different genes of the O-antigen biosynthetic gene cluster. We found that supplementation with exogenous CAI-1 or AI-2 caused a down regulation of these genes by 36% to 50% compared to control cultures without added exogenous autoinducers. Under the same conditions *luxO* gene was also down regulated whereas both *hapR* and *hapA* expression was significantly upregulated (Fig. 6). It may be mentioned that the activity of LuxO protein has been suggested to be regulated largely by phosphorylation, in which the phosphorylated form of LuxO activates the transcription of Qrr, which in turn acts as a repressor, and that the unphosphorylated form of the protein is inactive^17^,^23^. In the present study we found that the *luxO* gene is also transcriptionally regulated by autoinducers. Microarray based studies have been conducted previously to examine gene expression in *luxO* and *hapR* mutants^15^,^24^, although transcription of the *luxO* gene in response to added autoinducers in *V. cholerae* was not explored in these studies. However, the concept that *luxO* may be regulated at the transcriptional level has been previously recognized since in *V. harveyi, luxO* was shown to autorepress its own transcription through a feed back mechanism^25^.

### Pre-treatment in autoinducers diminishes bacterial agglutination with specific antiserum

To understand the effect of autoinducers on possible expression of the O1 antigen in *V. cholerae*, we directly investigated whether a proportion of *V. cholerae* O1 cells become negative for agglutination with specific polyclonal antiserum when grown in the presence of autoinducers. In this assay, bacteria were grown under differenct conditions and exposed to anti-O1 rabbit serum for 15 min in microcentrifuge tubes, and then centrifuged at low speed to sediment agglutinated bacteria while retaining non-agglutinated cells in the suspension phase. The proportion of total cells retained in the supernatant fluids was measured and taken to reflect the portion of the bacterial population that did not react robustly with the anti-O1 serum. We found that when grown in medium supplemented with spent medium of recombinant *E. coli* expressing autoinducers CAI or AI2, *V. cholerae* O1 cultures revealed a high proportion of cells that did not agglutinate with O1-antiserum (Table 2). Interestingly, this effect was not observed when mutant strains of C6706*lacZ* lacking specific receptors for the autoinducers were used (Table 2). We picked representative colonies from the plates used to quantitate cells that remained unagglutinated in these experiments and then tested them for reactivity with anti-O1 serum by slide agglutination. We found that more than 80% of the colonies were negative for agglutination with this antiserum suggesting that they had stable mutations or epigenetic alterations that eliminated O1 antigen expression.

## Discussion

The results described here have important implications in understanding phage-bacterial interactions and *V. cholerae* population structure both in cholera victims and in the aquatic environment. Several previous studies have suggested that seasonal cholera epidemics may end as a result of phage predation of the causative epidemic *V. cholerae* strains^1^,^2^,^3^. Since the *V. cholerae* strain responsible for the preceding epidemic often re-emerges and causes a subsequent epidemic during the next cholera season, it is obvious that sufficient number of the epidemic *V. cholerae* cells survive the phage attack by diverse ways. While the density of *V. cholerae* cells such as that occurs inside a cholera victim or in the environment during an ongoing epidemic of cholera may be a risk parameter for increased replication of phages within the bacterial population, our studies suggest that *V. cholerae* has evolved regulatory or genetic responces that minimize the risk of predation by phages under conditions of high cell density. Understandably, maintaining a persistently elevated defense strategy against phages is costly if phage resistance also intefers with properties needed for ecological fitness (e.g., host replication/colonization in the case of *V. cholerae*). In this study, we have identified several processes that elevate phage resistance within *V. cholerae* populations by exogenous (e.g., extracellular factors) as well as intrinsic (cell-associated factors) mechanisms.

Our data suggest that the strategy of using the quorum-regulated production of hemagglutinin protease (HAP) is an important factor in defense against phages within bacterial populations at high cell density. However, residual enhanced phage resistance exhibited by *hapA* negative strain (Fig. 4) in response to autoinducers indicates that other quorum-regulated factors also contribute to the process. Since mutations in genes for the O1 antigen biosynthesis caused the indicator *V. cholerae* strain to become resistant to the phages used in this study (Supplementary Table S3), we presume that these phages utilize the O1 antigen as their cell surface receptor. *V. cholerae* cells grown in the presence of autoinducers showed a dimished capacity to adsorb phages and to be agglutinated by anti-O1 serum (Table 2). Both phenotypes suggest that downregulation of O1 antigen phage receptor occurs in response to autoinducers and thus at high cell density. Consistent with this conclusion, we found that 10 different O-antigen biosynthetic genes were modestly downregulated on exposure to autoinducers (Fig. 6).

Growth in the presence of autoinducers also caused more cells to spontaneoulsy exhibit inheritable phage resistance. Although the precise mechanisms involved in this enhanced apparently stable phage resistance were not clear, these data suggest that autoinducers might promote some sort of epigenic change (e.g., a change in DNA methylation that might alter transcription) or a specific genetic alteration that could drive changes in O1 antigen expression. For example, we and our colleagues have previously demonstrated that production of the O1 antigen is subject to genetic ‘phase variation’ that occurs by slipped strand mispairing at homopolymeric nucleotide stretches in one of two genes (*wbeL* and *manA*) that are critical for O1 antigen biosynthesis^8^. Further work will be needed to understand whether exposure to autoinducers can specificallly alter the rate of slipped strand mispairing and thus selectively mutate genes that have evolved coding sequences that are hypersusceptible to mistakes made by error prone polymerases that might drive such phase variation phenomena.

A previous study demonstrated that mutants of *V. cholerae* O1 carrying transposon insertion in genes encoding cAMP or cAMP receptor protein (CRP) are more susceptible to predatory phages^26^ compared to the parent strains. Furthermore, it has been shown that CRP is also involved in biosynthesis of the autoinducer CAI-1 and thus modulates quorum sensing^27^. The enhanced phage susceptibility of the cAMP or CRP mutants may be explained as a consequence of possible inability of these strains to exhibit quorum-regulated phage resistance as described here.

While CAI-1 is narrowly distributed in *Vibrio* species^28^, AI-2 is produced by numerous bacterial species^28^,^29^,^30^. Therefore, AI molecules produced by vibrios or other bacterial species in the environment or by the normal intestinal microbiota present in humans or animals may also contribute to protecting *V. cholerae* from predation by phages. Clearly, the possibility that survival of phage predation by bacteria might also depend on bacterial interspecies communication should lead to further studies that might provide evidence for novel microbial interactions and cross-species phage resistance.

Bacteria employ various mechanisms to encounter the persistent threat of predation by phages^31^. These include the restriction endonucleases and adaptive immune systems such as CRISPR-Cas (clustered regularly interspaced short palindromic repeats/CRISPR-associated proteins) system^9^,^32^,^33^. However, bacteria in the environment frequently populate microbial communities called biofilms^14^,^15^, in which they exist in a state of high cell density. We propose that the quorum regulated phage resistance mechanisms described here may be a common phenomenon exhibited by biofilm-associated bacterial cells. Notably, recent studies has suggested that *E. coli* K-12 shows resistance to λ-phage when grown in the presence of *N*-acyl-L-homoserine lactones (AHL), a typical class of quorum sensing signal molecules used by many Gram negative bacteria^34^, and that in *V. anguillarum* quorum sensing determines the choice of antiphage defense strategy^35^. In the context of *V. cholerae*, a mechanism which reduces susceptibility of biofilm-associated cells to predatory phages would lead to the survival of a proportion of the epidemic *V. cholerae* cells after phage predation might collapse a cholera epidemic by eliminating planktonic cells. Presumably these surviving cells could seed the environment as a source of the pathogen for the next epidemic season once environmental conditions are once again favorable^1^,^2^,^3^. Furthermore, *V. cholerae* in the environment mostly exist in a dormant form which we have characterized and call conditionally viable environmental cells (CVEC)^10^,^16^. Laboratory preparation of CVEC-like cells requires formation of biofilms^10^ and hence it follows that CVEC in the environment may survive phage predation through the quorum-dependent phage resistance mechanisms described here. Within the environment, CVEC exist as clumps of dormant bacteria which remarkably can be resuscitated by exposure to quorum sensing autoinducers^16^. Thus, a complex AI-driven pathway may exist for the formation of *V. cholerae* CVEC cells that include their formation from cells that are transiently resistant to phage, their entry into a dormant state, and then their resuscitation under conditions which again induces transient phage resistance. Providing proof for such a hypothesis will be challenging given the difficulties inherent in studying an organism that cycles between the human host and the environment while modulating its dormancy and phage resistance.

In recent times there is a renewed interest in phages as an intervention tool against bacterial infections, an approach popularly known as phage therapy. We predict that the results of this study have relevance in phage therapy in enteric infections. Enteric bacterial pathogens including *V. cholerae* are known to colonize and multiply in the intestine, and thus exist in a high cell density state. Assuming that under such conditions the bacteria attain a state of enhanced phage resistance, the efficacy of phage-mediated intervention is likely to be significantly compromised. Hence in phage therapy, there may be a need to administer supplementary quorum modulators e.g., molecules that can serve as “quorum quenchers” to diminish the quorum mediated phage resistance. However, it may be mentioned that for certain pathogens such as *V. cholerae* O1, quorum quenching may cause enhanced virulence factor production^17^, and hence quorum modulators to be used need to be carefully chosen depending on the target pathogens. Nonetheless, the present study demonstrates that quorum sensing plays an important role in bacterial survival in the face of phage predation and thus has implications for improving phage-mediated interventions against bacterial infections.

## Materials and Methods

### Bacterial strains and phages

*V. cholerae* strains used as indicators in plaque assays or as hosts for phage preparations were either from clinical or environmental sources, and were available in our collection. Clinical strains were originally obtained from cholera patients who attended the hospitals of the International Centre for Diarrhoeal Disease Research, Bangladesh (icddr,b) located in Dhaka. Environmental *V. cholerae* strains and various phages were isolated from surface waters in Dhaka by routine sampling during 2002 to 2015. Among the environmental phages included in this study, JSF35 which grows on a wide range of *V. cholerae* O1 strains was isolated more recently and was found in abundance in surface water in Bangladesh during 2014 and 2015. The defined mutant strains were obtained from a previously described TnFGL3 insertion library of the El Tor biotype strain C6706*lacZ*^22^ or constructed in the present study. Different bacteria and phage strains included in the study are presented in Supplementary Table S1 and Table S4.

### Institutional approvals

All experimental protocols were approved by the Research Review Committee (RRC) and the Ethics Review Committee (ERC) of the icddr,b (Protocol numbers PR-15029 and PR-07018). All methods were conducted in accordance with the guidelines of the RRC and ERC. Informed consent was obtained for using any human samples, as directed in the ERC guidelines.

### Soft agar plaque assays

Generally, phage particles were quantified by the soft agar plaque assay using a suitable indicator strain as described previously^1^,^2^. For estimation of susceptibility of different *V. cholerae* strains to phages, logarithmic-phase cells (500 μl) of each strain grown under defined conditions as described later were mixed with 3.5 ml aliquots of soft agar (nutrient broth containing 0.8% Bactoagar, Difco), and 100 μl of the phage suspension containing approximately 3.2 × 10^2^ particles (pre-estimated using *V. cholerae* strain C6706*lacZ* as the indicator strain), and the mixtures were overlaid on nutrient agar plates. Plates were incubated for 16 h at 37 °C. Plaques were counted to estimate the level of phage susceptibility of the host strains. Variation in number of plaques produced with equal number of phage particles used indicated the level of phage susceptibility of the different strains when grown under different conditions.

### Screening of the transposon insertion library

A previously constructed TnFGL3 insertion library^22^ of the El Tor biotype strain C6706*lacZ*, was screened to identify mutants exhibiting altered susceptibility to different phages. The screening was done by cross streaking of the bacterial mutants and the phages on LB agar plates. The relevant mutants of C6706*lacZ* identified by cross-streaking were further confirmed by standard soft agar plaque assays as described above.

### Construction of mutants

Deletion mutants were prepared as described previously^16^. Briefly, appropriate primers were used to amplify two short DNA segments flanking the two ends of the targeted gene. These fragments were ligated to form a fusion product which was amplified further by PCR using forward primer of the left flanking fragment and the reverse primer of the right flanking fragment. The PCR product was cloned into the *Sma*I site of the suicide vector pRE112 and transformed into SM10λpir. Plasmid DNA isolated from selected colonies was used to electroporate appropriate recipient bacteria and plated on LB agar plates containing an antibiotic (20 μg/ml of chloramphenicol). The selected colonies were grown in LB with 5% sucrose for 3-4 h and plated on LB agar plates without any antibiotic. Colonies which lost the resistance marker and hence the suicide vector were further purified on LB-5% sucrose and their genotypes were confirmed by PCR.

For construction of defined double mutants, *V. cholerae* strains carrying a deletion mutation was further transformed with genomic DNA fragments of appropriate strains (carrying TnFGL3 inserts^22^ in the presence of chitin by using previously described methods^26^,^36^. Briefly, overnight cultures of the recipient *V. cholerae* strains were diluted 1:100 fold in LB medium, and grown to an OD_600_ of ~0.3. The bacteria were precipitated by centrifugation, washed and re-suspended in one tenth volume of filter-sterilized environmental water or 0.5% sterile sea salt solution (SS). Aliquots of a 2 ml bacterial suspension were dispensed into the wells of a 12-well tissue culture plate containing sterile pieces of shrimp-shell. After incubating statically at 30 °C for 24 hours, the planktonic phase was removed and fresh water or SS was added. At the same time, 1–2 μg of the appropriate DNA was added to the wells. After 24 h, the shrimp shells were removed from the wells, washed in SS, and vortexed in SS to release the attached bacteria. The released bacteria were then plated onto appropriate antibiotic-containing LB agar plates. Suspected transformants were further analyzed using PCR and hybridization assays to confirm the presence of TnFGL3 insertion markers in the relevant genes.

### Preparation of spent media containing autoinducers

The spent medium containing CAI-1 or AI-2 were prepared from cultures of *E. coli* DH5α carrying cloned *cqsA* gene encoding CAI-1 synthase or *luxS* gene encoding AI-2 synthase respectively. The recombinant constructs pJZ176 carried the *cqsA* gene whereas pJZ346 carried the *luxS* gene cloned in pTAC under an IPTG-inducible lac promoter as described previously^17^. For preparation of the spent medium, an overnight culture of the respective recombinant *E. coli* was diluted 1:100 times in fresh LB medium (100 mL) containing ampicillin (50 μg/mL), and grown for 3–4 h (until OD_600_  =  0.5–1.0). Cells were precipitated by centrifugation at 4,500 × g for 15 min, the supernatant was discarded, and the pellet was re-suspended in the original volume of fresh pre-warmed LB medium without any antibiotic. IPTG solution was added to a final concentration of 0.5 mM, and the culture was incubated for another 6–8 h at 37 °C with shaking. Aliquots of the culture were centrifuged at 6,000 × g for 20 min to precipitate bacterial cells. Culture supernatants containing the autoinducer were collected and sterilized by filtration through 0.22-μm pore-sized filters. Autoinducer concentrations in the culture supernatants were roughly estimated using bioluminescence-producing indicator strains MM920 and BB170 for CAI-1 and AI-2 respectively as described previously^15^,^37^,^38^, and comparing with known concentration of synthetic autoinducers.

### Phage-bacterial growth Kinetics

The dynamics of growth of *V. cholerae* strain C6706lacZ and its various mutants with each lytic phage was studied separately in a mixed culture of the respective phage and the bacteria in LB medium. The medium was inoculated simultaneously with a laboratory-grown bacterial culture and the phage preparation (diluted to an initial concentration of approximately 10^7^ bacteria and 10^6^ phage particles per ml), and was incubated at 37 °C with shaking. The initial counts of the bacteria and the phage were also verified later by culturing aliquots of the respective stock preparations obtained just prior to inoculating into the medium. Samples were removed from the mixed cultures at regular intervals and analyzed for the presence of phage and *V. cholerae* using standard plating techniques. Briefly, an aliquot of the culture was centrifuged at 4,500 × g for 15 min to precipitate bacterial cells. The supernatant was collected and filtered through 0.22 μm pore sized Millipore filter, and the filtrate was used to estimate phages by the soft agar plate assay as described above. The bacterial precipitate was re-suspended in fresh LB, and dilutions of the suspension were plated to determine bacterial counts. Before plating, the bacterial suspension was thoroughly vortexed to ensure that most cells including possible cellular aggregates if any were uniformly dispersed.

To study the effect of autoinducers on the phage bacterial growth kinetics, the medium was supplemented with 50% (v/v) spent medium prepared from recombinant *E. coli* over-expressing or not expressing the *V. cholerae* autoinducers CAI-1, and AI-2. Appropriate control assays were run in parallel to monitor the stability of the phage in the spent culture supernatants in the absence of the host strain.

### Estimation of phage-resistance

The frequency of emergence of phage resistant derivatives of different bacterial strains grown in the presence or absence of autoinducers was estimated using an assay described previously^21^. In brief, overnight culture of the *V. cholerae* strain was diluted 100 fold in fresh LB medium supplemented with 50% (v/v) spent culture medium of recombinant *E. coli* producing or not producing the autoinducers CAI-1 or AI-2 as described above, and grown to log phase. Bacterial cells were then collected by centrifugation, washed in LB to remove any residual protease. The cells suspended in LB were spread on a nylon membrane placed on an LB agar plate and incubated for approximately 6 h. When small colonies became visible, the nylon membrane was removed and placed upside down on a phage challenge plate (i.e., LB agar plate pre-inoculated with ~10^9^ phage particles) to obtain an impression of the colonies. The membrane was transferred back onto the original plate, and the sampling plate with the impression of the colonies, was incubated at 37 °C for 16 h. In this assay, phage-sensitive colonies either lysed or did not grow any further, whereas the phage-resistant colonies continued to grow and formed large colonies (Supplementary Fig. S2). To rule out the possibility that the apparent phage resistant colonies survived due to inactivation of phages by protease produced by the strain, representative colonies from the plate were picked, grown, and further confirmed for phage resistance by spotting a high titer of the phage on a lawn of the bacterium.

### Phage adsorption assay

Adsorption of different phages to *V. cholerae* O1 strain C6706*lacZ* grown in the presence or absence of autoinducers was studied following a previously described method with some modifications^25^,^39^. Briefly, overnight culture of the *V. cholerae* strain was diluted 100 fold in fresh LB medium supplemented with 50% (v/v) spent culture medium of *V. cholerae* expressing or not expressing cloned CAI-1 or AI-2 synthase gene as described above, and grown to log phase. To examine the adsorption of the phage to indicator bacterial cells, ~2.5 × 10^8^ cells in the broth culture of the *V. cholerae* strain was mixed with 10^7^ pfu of the phage. LB broth mixed with the phage without the bacteria was used as a control. Parallel samples were incubated for 20 min at 30 °C, and the concentrations of non-adsorbed phages were determined. To estimate the non-adsorbed phages in each sample, bacteria were removed by centrifugation, and supernatants were filtered through 0.22-μm-pore-size filters (Millipore Corporation, Bedford, MA,). Serial dilutions of the filtrate were plated on an indicator bacterial strain and plaque counts were determined after 16 h of incubation at 37 °C.

### GFP assay

Overnight culture of the strains carrying GFP reporter constructs were diluted 100 folds and grown for 6 h in LB containing 50% (v/v) spent medium prepared from recombinant *E. coli* cultures expressing cloned CAI-1 or AI-2, and from control *E. coli* cultures carrying the empty vector pTAC. Cells were precipitated, washed in PBS (pH 7.4), and re-suspended in equal volume of PBS. Fluorescence intensity was measured following a previously described method^40^. A 1:10 dilution of the suspension was prepared to photometrically determine cell densities at 600 nm. The ratio of fluorescence intensity to cell density was taken as a measure for GFP expression levels.

### Assay for Hemagglutinin protease

HA protease was assayed by its hemagglutination and protease activity. Microtiter quantitation of hemagglutination was conducted using chicken erythrocytes as described previously^41^. To test for inhibitory effects, various amounts of mannose, were added. Reactions were observed after incubation at room temperature for 30 min. Proteolytic activity was detected by using a single-diffusion technique in agar containing 1.5% skim milk (Difco Laboratories, Detroit, Mich.) as described previously^42^.

### Statistical analysis

General statistical analysis of data was done using the in-built data analysis program in Microsoft Excel (MS office version 2007). Data were expressed as mean ± standard deviation, and differences were tested by two-tailed t-test. The values P < 0.05 were considered statistically significant.

## Additional Information

**How to cite this article**: Hoque, M. M. *et al*. Quorum Regulated Resistance of *Vibrio cholerae* against Environmental Bacteriophages. *Sci. Rep.* **6**, 37956; doi: 10.1038/srep37956 (2016).

**Publisher's note:** Springer Nature remains neutral with regard to jurisdictional claims in published maps and institutional affiliations.

## Supplementary Material

Supplementary Information

## Figures and Tables

**Table 1:** Variation in number of plaques produced when equal aliquots of phage JSF35 (containing ~3.2 × 10^2^ particles)^a^ were plated on strain C6706*lacZ* and its derivatives.

Indicator Strain	Plaque counts with indicator bacteria grown in medium supplemented with spent culture supernatants of recombinant *E. coli* strains grown under different conditions^b^					
	LB	DH5α (pJZ176) with IPTG	DH5α (pJZ176) without IPTG	DH5α (pJZ364) with IPTG	DH5α (pJZ364) without IPTG	DH5α (pTAC) with IPTG
C6706*lacZ*	298 ± 15	47 ± 3	272 ± 12	48 ± 6	256 ± 12	275 ± 15
		p = 0.0084		p = 0.0002		
C6706*lacZ∆cqsA, luxS::TnFGL3*	296 ± 17	65 ± 7	286 ± 17	52 ± 5	271 ± 15	279 ± 18
		p = 0.0007		p = 0.0004		
C6706*lacZ, cqsS*::*TnFGL3*	257 ± 18	278 ± 11	258 ± 11	56 ± 5	279 ± 15	281± 16
				p = 0.0016		
C6706*lacZ*, *luxP*::*TnFGL3*	290 ± 6	63 ± 10	301 ± 17	296 ± 19	270 ± 22	290 ± 18
		p = 0.0034				
C6706*lacZ, luxO*::*TnFGL3*	38 ± 5	45 ± 7	35 ± 5	38 ± 3	30 ± 3	35± 7
C6706*lacZ* ∆*hapA*	282 ± 12	218 ± 15	279 ± 15	238 ± 15	285 ± 14	278 ± 15
		p = 0.0109		p = 0.0134		
C6706*lacZ* ∆*hapR*	291 ± 11	277 ± 12	292 ± 14	280 ± 10	291 ± 15	290 ± 11
C6706*lacZ*∆*hapA*, *luxO*::TnFGL3	193 ± 15	214 ± 19	227 ± 16	173 ± 19	238 ± 16	257 ± 18

^a^An approximate phage titer of ~3.2 × 10^3^/ml was determined initially using strain C6706*lacZ* as the indicator strain grown under standard conditions. Aliquots (100 μl) of this phage preparation were used for each assay. Variation in number of plaques produced with equal number of phage particles used indicated the level of phage susceptibility of the different strains when grown under the defined conditions.

^b^Values shown are mean and standard deviations of 3 independent observations. Relevant p values derived from two tailed t-test are shown under the mean values compared. In addition, p values for the difference in phage susceptibility of different mutants and parent strain without added autoinducers were C6706*lacZ, luxO*::TnFGL3 vs C6706*lacZ* (p = 0.0019); C6706*lacZ*∆*hapA* vs C6706*lacZ*∆*hapA*, *luxO*::TnFGL3 (p = 0.029) and C6706*lacZ* vs C6706*lacZ*∆*hapA*, *luxO*::TnFGL3 (p = 0.026).

**Table 2:** Percentage of bacterial cells non-agglutinating with polyclonal antiserum against *V. cholerae* O1 after pre-treatment under different conditions for 6 h.

Strain	Percentage of cells non-agglutinating with specific antisera after different bacterial strains were grown in medium supplemented with spent culture supernatant of recombinant *E. coli* strains carrying cloned AI synthase genes and grown under different conditions^a^					
	LB with IPTG	DH5α (pJZ176) with IPTG	DH5α (pJZ176) without IPTG	DH5α (pJZ364) with IPTG	DH5α (pJZ364) without IPTG	DH5α (pTAC) with IPTG
C6706*lacZ*	8.7 ± 0.3	47.6 ± 8.4	9.0 ± 0.7	52.2 ± 4.1	9.5 ± 1.4	10.2 ± 0.7
C6706*lacZ∆cqsA, luxS::TnFGL3*	7.5 ± 0.2	45.6 ± 3.7	8.0 ± 1.0	48 ± 2.5	8.2± 0.3	7.3± 0.2
C6706*lacZ* ∆*hapR*	8.8 ± 0.4	9.6 ± 1.4	10.1 ± 0.9	8.7 ± 1.1	9.4 ± 0.8	9.2 ± 1.0
C6706*lacZ* ∆*hapA*	5.9 ± 0.3	18.6 ± 1.6	5.9 ± 0.5	20.4 ± 0.6	5.3 ± 0.4	6.3 ± 0.5
C6706*lacZ*, *luxO*::TnFGL3	15.5 ± 1.4	16.4 ± 1.2	15.1 ± 0.8	14.2 ± 0.8	16.2 ± 0.9	14.8 ± 0.3
C6706*lacZ*∆*hapA*, luxO::TnFGL3	12.6 ± 1.2	13.2 ± 1.1	15.1 ± 0.6	15.2 ± 0.5	12.1 ± 0.9	13.2 ± 0.9
C6706*lacZ*∆*luxP*, cqsS::TnFGL3	10.0 ± 1.1	9.7 ± 0.9	12.3 ± 1.3	9.7 ± 0.5	9.4 ± 0.2	10.8 ± 0.6
O1-antigen negative phage resistant derivative of C6706*lacZ*	98.1 ± 1.5	98.1 ± 1.4	98.2 ± 1.4	98.1 ± 1.6	98.3 ± 1.8	96.2 ± 2.8
C6706*lacZ* assayed with O139-antiserum	94.2 ± 1.9	98.0 ± 1.2	96.6 ± 2.7	96.6 ± 2.7	98.3 ± 1.7	98.1 ± 1.2

^a^Bacterial strains were grown under specified conditions for 6 h, precipitated and re-suspended in fresh medium. The suspended bacteria were treated with specific polyclonal antiserum for 15 minutes and centrifuged at low speed for 2 min to precipitate agglutinated bacteria. The ratio of bacteria retained in suspension to total bacteria was expressed as percentage of cells non-reacting with the antiserum. Values shown are the mean and standard deviations of 3 independent observations.
