# Supplementary material for: Quorum Regulated Resistance of Vibrio cholerae against Environmental Bacteriophages
Source: Sci Rep. 2016 Nov 28;6:37956. doi: 10.1038/srep37956 (PMC5124996; doi:10.1038/srep37956)
Supplement: Supplementary Information [file srep37956-s1.pdf]

Supplementary Information

Quorum Regulated Resistance of *Vibrio cholerae* against Environmental Bacteriophages

M. Mozammel Hoque, Iftekhar Bin Naser, S. M. Nayeemul Bari, Jun Zhu,
John J. Mekalanos, and Shah M. Faruque*

Tables S1-S4

Figure S1

Figure S2

Table S1. Bacterial strains and phages used in this study.

Strain ID/genotype	Description	Source/Reference
C6706	*Vibrio cholerae* O1 El Tor biotype clinical isolate	Laboratory collection
C6706*lacZ*	Spontaneous *lacZ* negative derivative of C6706	Laboratory collection
C6706*lacZ, hapA::TnFGL3*	*V. cholerae* C6706*lacZ* carrying TnFGL3 insertion in *hapA* gene	20
C6706*lacZ, hapR::TnFGL3*	*V. cholerae* C6706*lacZ* carrying TnFGL3 insertion in *hapR* gene	20
C6706*lacZ,cqsS*::TnFGL3	*V. cholerae* C6706*lacZ* carrying TnFGL3 insertion in *cqsS* gene encoding CAI-1 sensor	20
C6706*lacZ*, *luxP*::TnFGL3	*V. cholerae* C6706*lacZ* carrying TnFGL3 insertion in *luxP* gene encoding AI-2 sensor	20
C6706*lacZ, luxO*::TnFGL3	*V. cholerae* C6706*lacZ* carrying TnFGL3 insertion in *luxO* gene	20
C6706*lacZ* Δ*hapA*	*hapA* deleted derivative of C6706*lacZ*	This study
C6706*lacZ* Δ*hapR*	*hapR* deleted derivative of C6706*lacZ*	This study
C6706*lacZ*Δ*hapA*, *luxO*::TnFGL3	*hapA* and *luxO* double mutant of C6706*lacZ*	This study
C6706*lacZ*Δ*luxP*	AI-2 sensor gene *luxP* deleted in C6706*lacZ*	This study
C6706*lacZ*Δ*luxP*, *cqsS*::TnFGL3	CAI-1 sensors gene *cqsS* inactivated in C6706*lacZΔluxP*	This study
E. coli DH5α (pJZ176)	Recombinant *E. coli* carrying *V. cholerae cqsA* gene cloned into plasmid *pTAC* to create *pJZ176*	17
E. coli DH5α (pJZ346)	Recombinant *E. coli* carrying *V. cholerae luxS* gene cloned into plasmid *pTAC* to create *pJZ176*	17
E. coli DH5α (pTAC)	*E. coli* carrying the empty cloning vector *pTAC*	17
JSF1, JSF2, JSF7, JSF10, JSF11, JSF16, JSF18, JSF23, JSF24, JSF25, JSF28, JSF31, JSF34, JSF35, JSF36	*V. cholerae* O1 specific bacteriophages isolated from environmental waters in Bangladesh	6
JSF3	*V. cholerae* O139 specific bacteriophage isolated from environmental water in Bangladesh	6

Table S2. Effect of spent culture supernatant of C6706*lacZ* and its derivatives on the stability of 15 different vibriophages of the JSF series (Faruque & Mekalanos, 2012).

Bacterial strain and growth condition	HA/ Protease activity[b]	[a]Stability (%) of different phages (ratio of plaque counts before and after treatment x 100) after incubation in culture supernatants[c] of different *V. cholerae* strains for 12h.														
		JSF1	JSF2	JSF7	JSF10	JSF11	JSF16	JSF18	JSF23	JSF24	JSF25	JSF28	JSF31	JSF34	JSF35	JSF36
LB medium	-/-	91±7.5	87±9.6	92±7.2	91±5.5	88±6.2	90±7.0	89±6.5	90±8.6	95±2.6	94±4.3	93±2.6	95±4.3	91±7.8	93±3.6	87±5.0
C6706*lacZ* /LB	-/-	68±3.6	78±5.2	92±2.6	76±3.6	92±3.4	70±4.3	82±3.4	81±2.6	86±4.3	78±4.5	89±3.6	87±4.5	77±5.2	82±4.5	70±5.5
C6706*lacZ* / LB with spent medium of IPTG- induced DH5α(pJZ176)	++/++	45±3.0	49±2.6	36±2.6	51±3.6	42±3.6	40±2.6	37±3.4	52±2.0	50±4.3	48±3.0	41±2.0	43±3.4	42±2.6	44±1.7	51±3.6
C6706*lacZ* / LB with spent medium of IPTG- induced DH5α(pJZ364)	++/++	47±3.0	51±2.3	42±2.6	45±1.0	42±3.0	42±2.6	39±3.0	45±2.6	52±4.3	50±2.0	45±2.0	39±1.7	36±3.4	47±2.8	50±3.6
C6706*lacZ* / LB with spent medium of IPTG induced DH5α(pTAC)	-/-	70±5.5	77±4.5	89±3.6	82±5.1	90±5.2	72±6.0	79±7.5	87±3.6	83±4.3	82±4.0	90±3.6	84±2.6	80±5.5	79±5.0	75±4.3
C6706*lacZ* ΔhapA	-/-	70±4.5	76±2.6	92±2.6	72±3.0	88±7.8	69±5.5	85±6.2	80±3.4	84±4.0	70±5.0	85±5.5	80±5.2	72±6.0	79±4.5	65±2.5
C6706*lacZ* ΔhapR	-/-	71±2.6	80±5.2	89±5.5	77±4.7	94±3.0	77±4.0	90±3.6	87±3.0	90±6.2	75±6.0	84±5.1	82±6.5	79±7.0	85±3.2	71±5.5
C6706*lacZ*,luxO::TnFGL3	+++/+++	40±2.6	42±3.0	28±2.0	41±1.7	40±2.6	42±3.6	34±2.0	42±2.0	42±2.6	46±3.6	35±3.0	34±2.6	40±2.6	34±2.0	39±2.0
C6706*lacZ* ΔhapA luxO::TnFGL3	-/-	68±3.0	72±2.3	88±2.0	82±2.8	79±6.4	70±3.2	82±4.5	78±3.0	80±3.2	75±6.0	79±3.6	75±2.5	68±2.0	78±3.6	64±3.6

[a]Values shown are mean and standard deviations of 3 independent observations; fractions have been rounded up to whole numbers.

[b]All strains except the ones indicated otherwise were grown in LB medium. Hemagglutination and protease activities are semi-quantitative estimate based on the extent of agglutination of chicken erythrocytes, and the zone size of protease activity on skimmed milk agar plates respectively.

[c]Culture supernatants were prepared after 4h growth when wild type strain C6706*lacZ* did not express HAP under normal culture conditions, but expressed HAP in the presence of added AIs.

Table S3: Sensitivity of mutants with transposon insertions in O-antigen biosynthesis genes of *V. cholerae* O1 strain C6706*lacZ* to 15 different vibriophages of the JSF series (Faruque & Mekalanos, 2012).

Description of strains	Sensitivity to various phages of the JSF series														
Phage designations	1	2	7	10	11	16	18	23	24	25	28	31	34	35	36
C6706*lacZ*	S	S	S	S	S	S	S	S	S	S	S	S	S	S	S
C6706*lacZ*, *manC*::TnFGL3	**R**	**R**	S	S	**R**	S	S	**R**	**R**	**R**	**R**	**R**	**R**	**R**	**R**
C6706*lacZ*, *manB*::TnFGL3	**R**	**R**	S	S	S	S	S	S	S	S	**R**	**R**	**R**	**R**	**R**
C6706*lacZ*, *wbeE*::TnFGL3	**R**	**R**	S	S	**R**	S	S	S	**R**	**R**	**R**	**R**	**R**	**R**	**R**
C6706*lacZ*, *wbeG*::TnFGL3	**R**	**R**	S	S	S	S	S	S	**R**	S	**R**	**R**	**R**	**R**	**R**
C6706*lacZ*, *wbeH*::TnFGL3	**R**	**R**	S	S	**R**	S	S	**R**	**R**	**R**	**R**	**R**	**R**	**R**	**R**
C6706*lacZ*, *wbeI*::TnFGL3	**R**	**R**	S	S	**R**	S	**R**	**R**	**R**	**R**	**R**	**R**	**R**	**R**	**R**
C6706*lacZ*, *wbeL*::TnFGL3	**R**	**R**	S	S	**R**	S	S	**R**	**R**	**R**	**R**	**R**	**R**	**R**	**R**
C6706*lacZ*, *wbeU*::TnFGL3	**R**	**R**	S	S	S	S	S	**R**	**R**	S	**R**	**R**	**R**	**R**	**R**
C6706*lacZ*, *wbeV*::TnFGL3	**R**	**R**	S	S	S	S	S	**R**	S	S	**R**	**R**	**R**	**R**	**R**
C6706*lacZ*, *gmd*::TnFGL3	S	S	S	S	S	S	S	S	S	S	S	S	S	S	S
C6706*lacZ*, *wbeT*::TnFGL3	S	S	S	S	S	S	S	S	S	S	S	S	S	S	S

S = susceptible; R = resistant

[6]Faruque SM, Mekalanos JJ. 2012. Phage bacterial interactions in the evolution of toxigenic *Vibrio cholerae*. Virulence 3:1-10

Table S4. Mutants of C6706*lacZ* carrying Tn insertions in O-antigen biosynthetic genes obtained from a transposon insertion library (Cameron et al., 2008) used in this study

Gene Locus	Description/gene product	Gene name
VC0241	mannose-1-phosphate guanylyltransferase	*manC*
VC0242	phosphomannomutase	*manB*
VC0243	GDP-mannose 4,6-dehydratase	*gmd*
VC0244	perosamine synthase	*wbeE*
VC0245	rfbG protein	*wbeG*
VC0246	lipopolysaccharide/O-antigen transport protein	*wzm*
VC0247	lipopolysaccharide/O-antigen transport protein	*wzt*
VC0249	rfbL protein	*wbeL*
VC0258	rfbT protein	*wbeT*
VC0259	lipopolysaccharide biosynthesis protein	*wbeU*
VC0260	mannosyl-transferase	*wbeV*
VC0269	Mannose-6-phosphate isomerase	*manA*

[20]Cameron DE, Urbach JM, Mekalanos JJ. 2008. A defined transposon mutant library and its use in identifying motility genes in *Vibrio cholerae*. Proc. Natl. Acad. Sci. USA, 105:8736–8741.

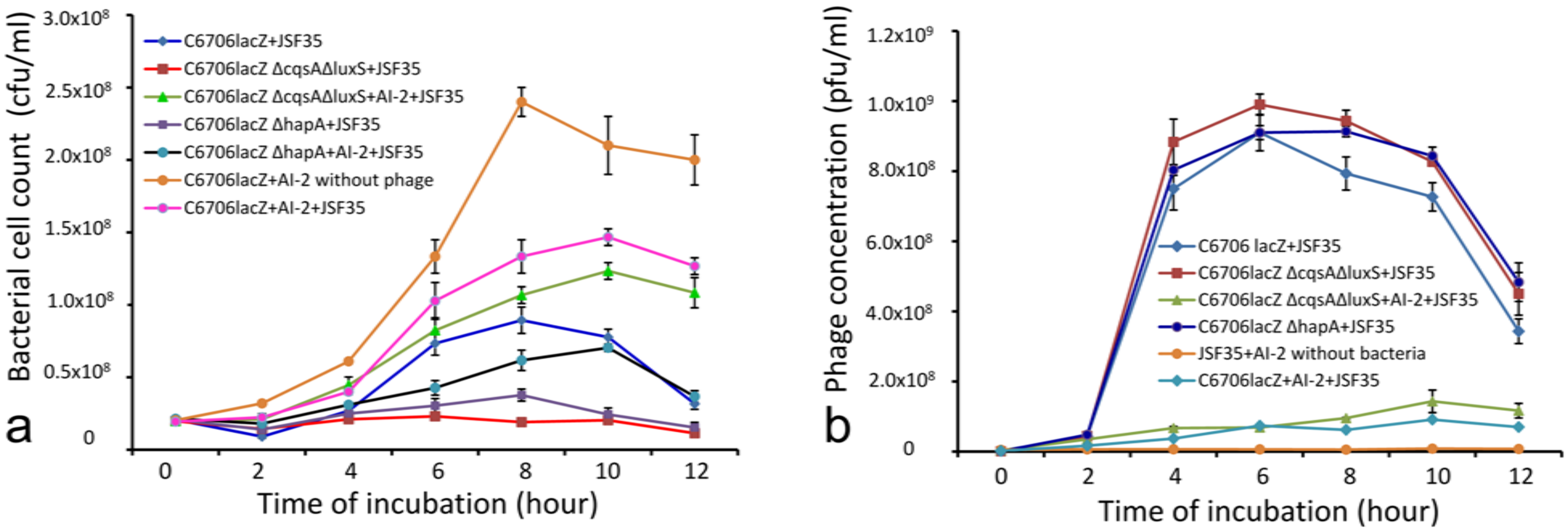

Figure S1. Effect of exogenous autoinducer AI-2 on phage-bacterial growth kinetics in mixed cultures of a phage and a susceptible *V. cholerae* strain or its derivatives. LB medium was supplemented with 50% (v/v) spent medium of recombinant *E. coli* over-expressing or not expressing the *V. cholerae* autoinducer AI-2. The medium was inoculated simultaneously with a laboratory-grown bacterial culture and the phage preparation (diluted to a concentration of ~10^7 bacteria and 10^6 phage particles per ml), and was incubated at 37°C with shaking. Samples were then removed at regular interval and analyzed for the presence of phage and *V. cholerae* using standard plating techniques. Appropriate control assays were run in parallel to monitor the stability of the phage in the spent culture supernatants in the absence of the host strain. (a) Growth curve of *V. cholerae* strain C6706*lacZ* and its derivatives with a lytic phage JSF35 in the presence or absence of exogenous *V. cholerae* autoinducer AI-2 as indicated; (b) Titer of phage JSF35 subjected to different growth conditions and host strains as indicated. Each data point represents the mean and standard deviation of three observations.

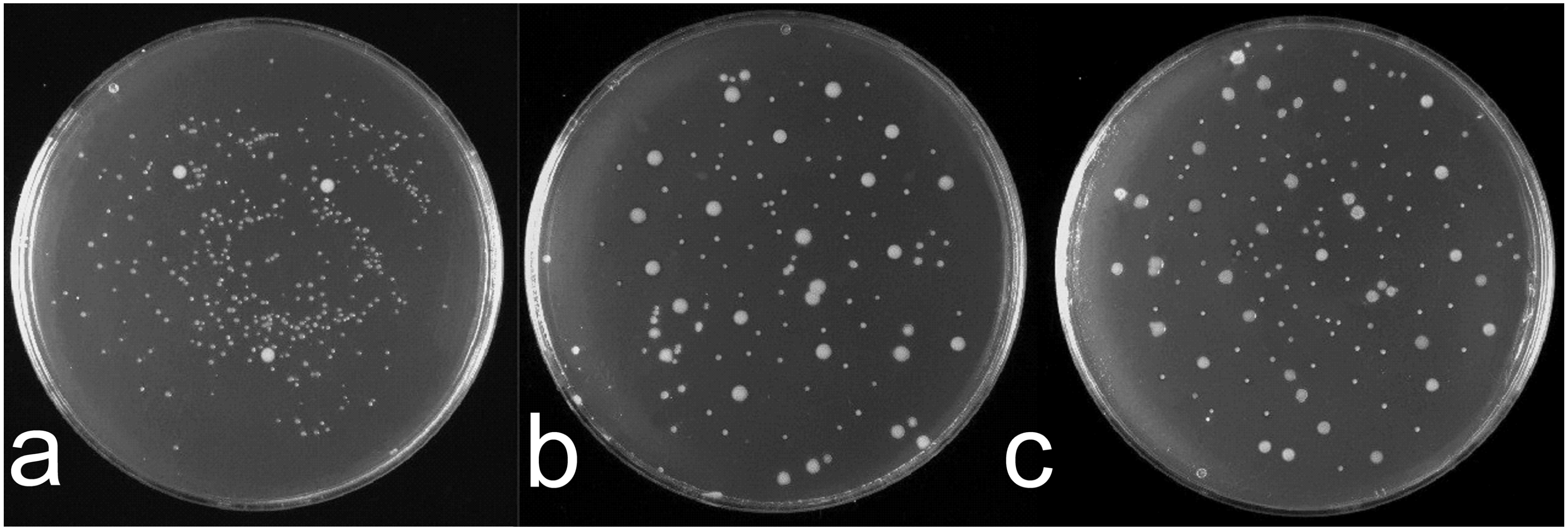

Figure S2. Plate assay for estimation of the emergence of phage-resistant *V. cholerae*. An overnight culture of *V. cholerae* strain C6706*lacZ* was diluted 100 fold and grown for 6h in LB supplemented with 50% vol:vol spent culture medium from recombinant *E. coli* strains over-expressing or not expressing cloned CAI-1 or AI-2. *V. cholerae* cells were collected by centrifugation, washed in LB to remove any residual protease, and then spread on a nylon membrane placed on an LB agar plate and incubated for approximately 6h. When small colonies became visible, the nylon membrane was removed and placed upside down on a phage challenge plate (i.e., LB agar plate pre-inoculated with ~10^9 JSF35 phage particles) to obtain an impression of the colonies. The membrane was transferred back onto the original plate, and the sampling plate with the impression of the colonies, was incubated at 37°C for 16 h. In this assay, phage-sensitive colonies either lysed or did not grow any further, whereas the phage-resistant colonies continued to grow and formed large colonies (Fig. S1). Plates show bacterial colonies derived from growth in medium supplemented with IPTG induced spent culture supernatant of (a) *E. coli* DH5α carrying the empty cloning vector (pTAC); (b) *E. coli* DH5α (pJZ364) containing AI-2; and (c) *E. coli* DH5α (pJZ176) containing CAI-1
